# Healthcare system disruption across WHO-declared PHEICs: a 4S comparative case analysis

**DOI:** 10.3389/fpubh.2026.1901344

**Published:** 2026-09-14

**Authors:** Jung Min Kim, Jae Min Lee, Hye Ok Kim, Youn-Jung Kim, Sung Soo Kim, Manish Kumar Singh, Jin Woo Choi, Seung Geun Yeo

**Affiliations:** 1 Department of Otorhinolaryngology – Head and Neck Surgery, College of Medicine, Kyung Hee University Medical Center, Kyung Hee University, Seoul, Republic of Korea; 2 Department of Medicine, College of Medicine, Kyung Hee University, Seoul, Republic of Korea; 3 Clinical Research Institute, Kyung Hee University Medical Center, Seoul, Republic of Korea; 4 College of Nursing Science, Kyung Hee University, Seoul, Republic of Korea; 5 Department of Biochemistry and Molecular Biology, College of Medicine, Kyung Hee University, Seoul, Republic of Korea; 6 Department of Pharmacology, College of Pharmacy, Kyung Hee University, Seoul, Republic of Korea

**Keywords:** comparative analysis, emergency preparedness, health system capacity, health system resilience, healthcare disruption, public health emergency of international concern

## Abstract

**Objectives:**

To compare documented patterns of healthcare-system disruption and response across WHO-declared Public Health Emergencies of International Concern (PHEICs) using the established 4S surge-capacity framework (Staff, Stuff, Space, Systems) and an author-developed 12-indicator operationalization.

**Methods:**

We conducted a structured comparative case analysis, not a systematic review of intervention effects, of all eight PHEIC events declared by WHO through 31 March 2026. The unit of analysis was the event, and the coded observations were operational occurrences documented in official sources. Twelve binary, unweighted author-developed indicators, three per 4S domain, were mapped to the four established domains. Activation required either one primary official source or two independent secondary sources meeting a prespecified threshold. Two reviewers independently assigned indicator-level codes, with disagreements resolved by consensus; indicators classified as Not Reported were combined with Not Activated. Activated indicators were summed within domains (range 0–3), and each observed configuration was assigned to one of four profiles using a fixed hierarchical rule sequence.

**Results:**

All eight events activated at least one indicator, and external workforce mobilization (S1) and diagnostic bottleneck (T3) were documented in every event. COVID-19 was the only event activating all 12 indicators (systemic convergence). The two Ebola virus disease events shared an identical configuration of complete Staff, Stuff, and Systems activation with Space limited to temporary or repurposed facilities (workforce-cascade). H1N1 influenza, poliomyelitis, and mpox clade I formed the residual partial multi-domain category and did not share a common domain signature. Zika virus disease and mpox clade II met the limited-domain rule, with no Space-domain activation.

**Conclusion:**

Public Health Emergencies of International Concern designation was not sufficient to identify a common pattern of documented health-system disruption. The typology is a provisional descriptive partition rather than a severity ranking, causal model, or predictive tool, and its residual category is heterogeneous by construction. Profiles are conditional on the documentary evidence base and the settings it represents; baseline health-system capacity was not incorporated, so the same pathogen could plausibly yield a different configuration elsewhere. The framework may offer a common vocabulary for discussing domain-specific preparedness needs under the revised International Health Regulations and the developing WHO Pandemic Agreement, but external validation is required.

## 1 Introduction

A Public Health Emergency of International Concern (PHEIC) is a legal designation under the International Health Regulations used by the World Health Organization (WHO) to identify extraordinary public health events that may pose a risk to other countries through international spread and require a coordinated international response (1). Unlike the generic term “pandemic,” which lacks a universal legal definition, a PHEIC declaration constitutes a formal institutional mechanism for coordinated international public health action (2). Although not all PHEIC events escalate to global pandemics—with several remaining geographically concentrated despite imposing severe structural pressure on affected health systems—this formal designation provides a standardized, predefined case frame for comparing major international health emergencies.

Declared PHEIC events encompass pathogens with different transmission routes and implications for healthcare delivery. These include highly transmissible respiratory viruses, such as H1N1 influenza and COVID-19 (3, 4); diseases transmitted through direct contact with infected bodily fluids, lesions, or contaminated care environments, such as Ebola virus disease and mpox (5, 6); vector-borne infections, such as Zika virus disease (7); and vaccine-preventable infections with cross-border outbreak risk, such as poliomyelitis. The burden imposed by these events is not captured fully by aggregate case counts or mortality rates. Health system strain depends on interactions among pathogen characteristics, care requirements, transmission risks in clinical settings, and baseline system capacity (8). Although between-country differences in capacity limit direct comparison of impact magnitude, recurring patterns of functional disruption can still be compared across events.

Different pathogens may place pressure on different components of a health system. Respiratory transmission may increase demand for isolation, oxygen, critical care, diagnostics, and infection-control supplies (9, 10), whereas strict contact precautions may place particular demands on workforce availability, staff safety, training, and continuity of essential services (11). Vector-borne or geographically concentrated events may produce more localized or service-specific effects. These contrasts motivate an exploratory comparison of where disruption and response were documented across the 4S domains. Transmission route and care requirements are used only as contextual descriptors; this study does not test pathogen-class effects.

Previous assessments of PHEIC events have often emphasized event-specific epidemiology, clinical severity, or response chronology (12, 13). While recent systematic reviews have documented the broad impacts of PHEIC events on routine disease control programs (14), comparative analyses utilizing a standardized framework to assess the distribution of disruption across core health system functions remain limited. Consequently, it remains undetermined whether diverse PHEICs converge on common structural vulnerabilities or generate distinct disruption profiles dictated by transmission routes, clinical care pathways, and baseline system capacity.

The analytical lens used here is the 4S surge-capacity framework, which is adopted from existing literature rather than developed *de novo* in this study. The framework originates in disaster medicine, where surge capacity was decomposed into its essential constituent elements—personnel, materiel, and physical structure—by Barbisch and Koenig (15) and by Kaji et al. (16), and was subsequently extended to include the system-level arrangements that coordinate those elements, most influentially in the graded surge-capacity taxonomy of Hick et al. (17) and in the CHEST consensus statement on surge capacity principles (18). The resulting “Staff, Stuff, Space, and Systems” formulation has since been used as an operational shorthand in emergency and humanitarian health planning (19), and it can be conceptually aligned with selected components of the WHO health-system building blocks (20). What the existing literature does not provide is a set of comparable, externally documentable indicators that would allow the framework to be applied uniformly across separate international emergencies; the 4S construct has typically been used descriptively within a single event or institution.

This study therefore adopts the established 4S domains unchanged and develops an author-defined 12-indicator coding instrument for application across eight WHO-declared PHEIC events. Three analytic layers follow from the instrument—indicator-level activation, domain-level activation counts, and a rule-based descriptive partition—and their relationships are set out in Section 2.2 and Figure 1. The resulting profiles are not empirically derived latent classes and should be interpreted as a provisional organization of the observed configurations. The study asks whether the documented events exhibit recurring patterns across the four domains and how such patterns might inform preparedness planning. Transmission route and care requirements are treated only as contextual descriptors.

**Figure 1:**
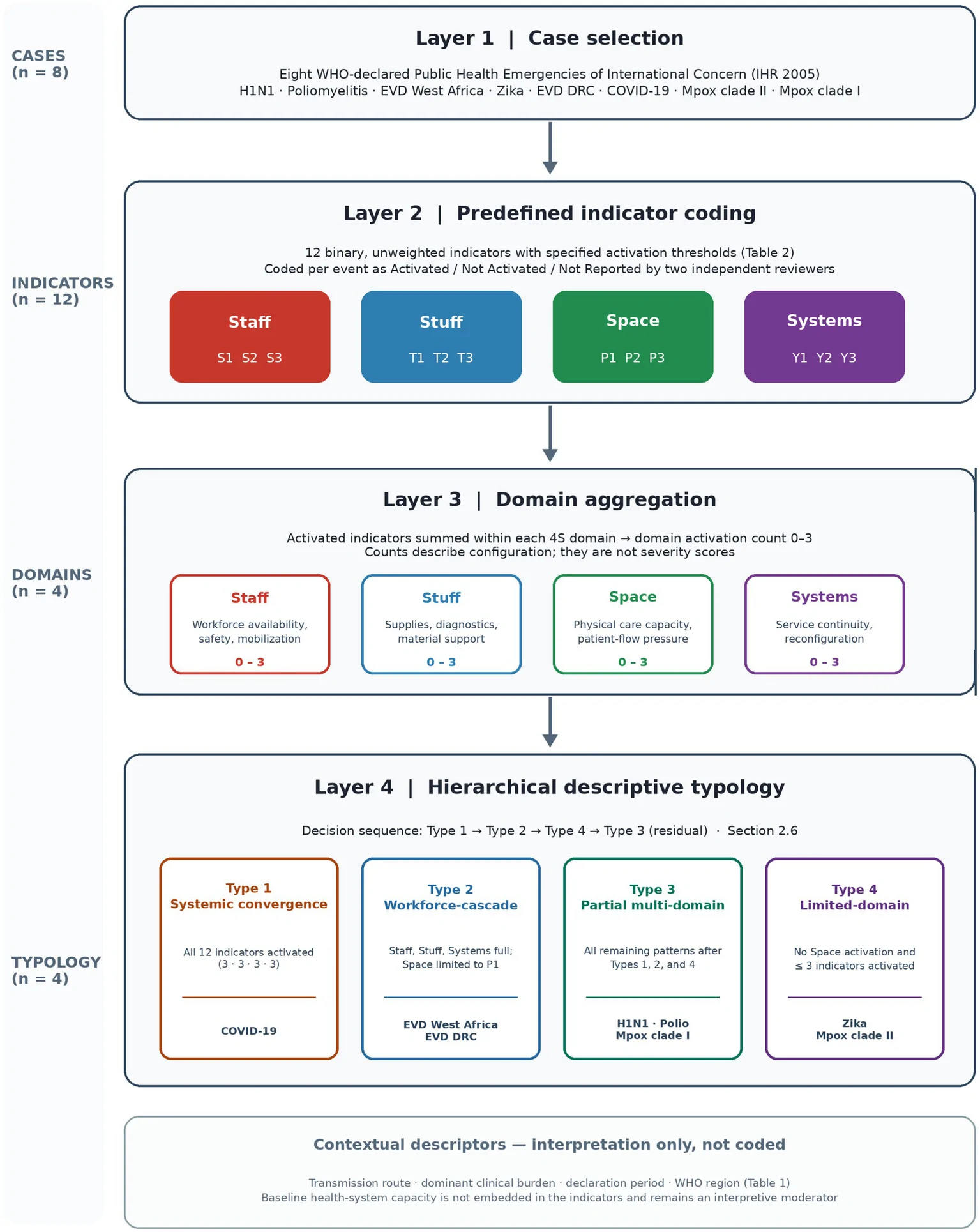
Relationships among the analytic layers of the 4S comparative framework. Layer 1 comprises all eight WHO-declared PHEIC events included in the date-defined analytic cohort through 31 March 2026. Twelve predefined binary indicators are coded per event against the thresholds in Table 2 and assigned to one 4S domain each. Activated indicators are summed within domains to yield counts of 0–3. Profiles are then assigned using the hierarchical decision sequence Type 1 → Type 2 → Type 4 → Type 3 (residual), as specified in Section 2.6. Contextual descriptors are used for interpretation only and do not enter the coding chain.

## 2 Methods

### 2.1 Study design

This study is classified as a structured comparative case analysis supported by a structured documentary evidence-identification process. It is not a systematic review of intervention effects or study findings: the unit of analysis is the PHEIC event, and the coded observations are operational occurrences, such as a mandated suspension of elective surgery, establishment of field treatment facilities, or formal restriction of testing. Evidence of these occurrences resides principally in WHO situation reports and Disease Outbreak News, national and subnational health authority directives, and operational reports from international and regional agencies. Peer-reviewed studies were used mainly to corroborate or date events identified in official sources.

Because the analysis did not estimate pooled effects, synthesize study-level outcomes, or assess certainty of evidence, a conventional systematic-review flow diagram and risk-of-bias framework were not used. Nevertheless, the databases and repositories searched, core search structure, eligibility criteria, source hierarchy, coding thresholds, and dual-review procedures are reported in Supplementary File 1. Indexed database searches were used to reduce omission of supporting published evidence, while official-document and grey-literature searches constituted the principal evidence-gathering activity. The synthesis is configurational rather than aggregative (21), and the findings should be interpreted as a structured comparison of documented events rather than pooled evidence about intervention effects.

The eight cases comprise all PHEIC events declared by WHO under the International Health Regulations through 31 March 2026. PHEIC status was defined according to official declarations issued under the International Health Regulations (1): H1N1 influenza, poliomyelitis, Ebola virus disease in West Africa, Zika virus disease, Ebola virus disease in the Democratic Republic of the Congo, COVID-19, mpox clade II, and mpox clade I. No case-level inclusion or exclusion criteria were applied. The unit of analysis was the PHEIC event. Indicator coding drew on event-specific documentation from one or more affected jurisdictions represented in WHO, national, and regional sources. Because the geographic scope of the documentary evidence differed across events, some event profiles were informed mainly by geographically concentrated outbreak settings, whereas others incorporated evidence from multiple affected settings. The resulting codes therefore characterize documented event-level configurations within the available evidence base and should not be interpreted as standardized estimates for a single country or jurisdiction.

Table 1 provides contextual characteristics, including the official declaration period, primary transmission route, dominant clinical burden, and the WHO region or regions represented in the documentary evidence base. These variables were not included in indicator or profile assignment and do not adjust for baseline health-system capacity. Health-system disruption and response were evaluated separately using the 4S framework described below.

**Table 1:** Key epidemiological characteristics of the eight WHO-declared PHEIC events.

Event	Declaration period	Primary transmission route	Dominant clinical burden	WHO region represented in the evidence base
H1N1 Influenza	Apr 2009 – Aug 2010	Respiratory (Droplet/Airborne)	Acute respiratory failure	Region of the Americas
Poliomyelitis	May 2014 – ongoing	Fecal-oral	Irreversible acute flaccid paralysis	Eastern Mediterranean/African Region
Ebola virus disease (West Africa)	Aug 2014 – Mar 2016	Direct contact (bodily fluids)	Profound hemorrhagic fever and shock	African Region
Zika virus disease (Americas)	Feb 2016 – Nov 2016	Vector-borne (Aedes)/Sexual	Congenital microcephaly and GBS	Region of the Americas
Ebola virus disease (DRC)	Jul 2019 – Jun 2020	Direct contact (bodily fluids)	Profound hemorrhagic fever and shock	African Region
COVID-19	Jan 2020 – May 2023	Respiratory (Airborne/Droplet)	Severe pneumonia and ARDS	Multiregional
Mpox Clade II	Jul 2022 – May 2023	Direct contact/Sexual	Disseminated painful dermal lesions	Region of the Americas/European Region
Mpox Clade I	Aug 2024 – Sep 2025	Direct contact	Severe systemic dermal lesions	African Region

ARDS, acute respiratory distress syndrome; GBS, Guillain–Barré syndrome; PHEIC, Public Health Emergency of International Concern; WHO, World Health Organization.

### 2.2 Conceptual framework and relationships among the analytic layers

Because the analysis moves through several nested classifications, their provenance and relationships are stated explicitly before the coding procedure. The analysis has four layers, summarized in Figure 1: eight cases, 12 indicators, four domains, and four disruption profiles. Indicator codes are aggregated into domain counts, after which a hierarchical rule set assigns each observed configuration to one profile. Contextual descriptors are used only for interpretation.

The four domains are adopted from the established 4S surge-capacity framework described in Section 1 (15, 17, 18) and are not a contribution of this study. The 12 indicators are author-developed operationalizations of those domains. Exactly three indicators were assigned to each domain so that all domain activation counts share a common 0–3 range; this symmetry was imposed for comparability and does not imply that the domains contain equal numbers of underlying dimensions or that the indicators are equally important. An indicator was retained if it could be assigned to one domain, documented across heterogeneous events and eras, and linked to a threshold stated without reference to a particular event. The set samples recurrent, externally documentable manifestations of strain or adaptive response; it is neither exhaustive nor externally content-validated. Binary coding was used because documentary granularity differed substantially between events, making an ordinal or weighted scale difficult to defend (21).

The four disruption profiles are not an external framework but a hierarchical, rule-based partition of the domain activation patterns, defined in Section 2.6. Rules are applied in the following order: Type 1, Type 2, Type 4, and finally Type 3 as the residual category. This sequence makes the assignments mutually exclusive and jointly exhaustive. Type 3 therefore contains the remaining observed configurations after the more specific Type 1, Type 2, and Type 4 rules have been applied; its members are not assumed to be internally homogeneous. Transmission route and clinical burden sit outside this coding chain and enter only at the interpretation stage.

### 2.3 Analytical framework: operationalizing the 4S model

We operationalized the 4S model into 12 indicators to compare documented health-system disruption and response across PHEIC events. The four domains were defined as follows: Staff (workforce availability, safety, and mobilization); Stuff (supplies, diagnostics, and material support); Space (physical care capacity and patient-flow pressure); and Systems (continuity of non-target services and system-level reconfiguration). Candidate indicators were selected through a structured review of WHO technical guidance and outbreak-response literature and finalized before profile assignment. An indicator was retained if it reflected a distinct, externally documentable occurrence and could be linked to a threshold in Table 2. Local operational adjustments without evidence of regional, national, or international escalation were not coded as activated. The instrument was designed for cross-event comparability rather than as a comprehensive or validated scale of health-system performance.

**Table 2:** Operational definitions of the 12 indicators in the 4S framework.

Domain	Code	Indicator	Operational definition
**S**taff	S1	External workforce mobilization	Official deployment of military medical personnel, retired healthcare workers, or out-of-jurisdiction volunteers to frontline duties
	S2	International medical teams	Formal request for, or confirmed deployment of, WHO Emergency Medical Teams (EMTs) or foreign governmental medical missions
	S3	HCW infection disruption	Official reports of ward closures, service suspensions, or critical staffing shortages directly attributed to healthcare worker infection clusters
S**t**uff	T1	Core supply shortage	National or regional health authority declarations reporting critical depletion of PPE, supplemental oxygen, ventilators, or essential therapeutics
	T2	International material aid	Formal activation of international emergency procurement mechanisms or documented receipt of foreign medical logistics
	T3	Diagnostic bottleneck	Official guidance restricting testing to high-priority groups, or documented laboratory turnaround times exceeding 72 h for over 1 week
S**p**ace	P1	Temporary facilities	Activation of field hospitals or the repurposing of non-clinical infrastructure (e.g., stadiums, convention centers) for patient care
	P2	ICU saturation	Official health authority reports indicating regional or national critical care occupancy exceeding routinely available baseline capacity
	P3	Patient transfer overflow	Implementation of structured inter-regional or international patient transfer protocols specifically due to local bed shortages
S**y**stems	Y1	Suspension of elective surgery	National or regional government mandates officially halting non-emergent elective surgical procedures
	Y2	Outpatient restriction	Official directives mandating the closure of routine ambulatory clinics or the mandatory transition to telemedicine for non-urgent care
	Y3	Non-target service disruption	Documented interruption of routine public health programs (e.g., immunizations, maternal care, HIV/TB management) explicitly attributed to the outbreak response

HCW, healthcare worker; HIV, human immunodeficiency virus; ICU, intensive care unit; PPE, personal protective equipment; TB, tuberculosis; WHO, World Health Organization.

For each PHEIC event, two researchers independently assigned each indicator to one of three categories: “Activated,” “Not Activated,” or “Not Reported.” “Activated” required documentation meeting the threshold in Table 2. “Not Activated” was used when relevant sources addressed the domain but did not meet the threshold, whereas “Not Reported” indicated insufficient information. Disagreements were resolved by consensus after review of the available source material. For the binary matrix and profile assignment, only “Activated” was treated as present; “Not Activated” and “Not Reported” were both displayed as non-activation. This rule avoids counting undocumented occurrences as activated but may bias less extensively documented events toward lower activation counts.

### 2.4 Search strategy and data sources

Data extraction followed a structured search protocol supporting event-level comparison. PubMed, Scopus, and Web of Science were searched from the official declaration date of each PHEIC through 31 March 2026 using event-specific terms combined with health-system keywords, including “surge capacity,” “workforce depletion,” “healthcare worker infection,” “supply shortage,” “diagnostic capacity,” “ICU capacity,” “elective surgery,” and “service disruption.” Supplementary File 1 reports the core Boolean structure, repositories searched, source hierarchy, eligibility criteria, and activation requirements. Because coding rested principally on official documentation rather than on study findings, the database search served as a check against omission of relevant published evidence rather than as the primary evidence-gathering step.

Grey literature searches were conducted in the official repositories of international and regional health organizations, including WHO, PAHO, and Africa CDC, and in national health ministry sources when necessary to assess jurisdiction-specific measures. Eligible documents included WHO situation reports, national health authority directives, international agency reports, and peer-reviewed observational studies describing observed health system strain or service reconfiguration. Documents published in English, French, or Spanish were eligible. Predictive modeling studies without observed health system data, clinical trials limited to individual-level outcomes, and editorials or commentaries lacking primary operational evidence were excluded.

Official WHO situation reports and national health ministry directives served as the principal sources for indicator coding. International agency reports and peer-reviewed observational studies supplemented these sources when they provided explicit evidence corresponding to predefined indicator thresholds. In cases of discrepancy, priority was given to official jurisdiction-specific documents over secondary summaries.

### 2.5 Data extraction and coding procedures

Each PHEIC event was evaluated using the 12-indicator 4S matrix and the thresholds in Table 2. During extraction, each indicator was assigned as “Activated,” “Not Activated,” or “Not Reported.” Isolated local shortages and routine outbreak-control activities were not coded as activated unless they met the specified level of escalation. Because the geographic scope of the documentary evidence base varied from concentrated outbreak settings to multiregional evidence, indicator activation represents threshold-level documentation within the event-specific evidence base rather than a standardized national or jurisdiction-level estimate.

An indicator was coded as “Activated” when threshold-level documentation was identified in at least one primary official source or in at least two independent secondary sources. Two reviewers independently screened the available documents and assigned indicator-level codes; discrepancies were resolved by consensus. Final event-level codes were retained in the analysis matrix. For the binary matrix used to generate domain counts and profiles, “Not Reported” was combined with “Not Activated” as non-activation. No weighting or severity scaling was applied. Accordingly, activation represents the documented presence of a threshold-level occurrence, not its intensity, duration, geographic extent, or population-level magnitude.

### 2.6 Data analysis

After construction of the 4S matrix, activated indicators were summed within each domain, yielding counts from 0 to 3. These counts describe configuration and were not interpreted as severity scores. Profiles were assigned using the following hierarchical rules: Type 1 (Systemic convergence), all 12 indicators activated (Staff 3; Stuff 3; Space 3; Systems 3); Type 2 (Workforce-cascade), Staff, Stuff, and Systems fully activated with Space limited to P1 (Staff 3; Stuff 3; Space 1; Systems 3; P2 = 0; P3 = 0); Type 4 (Limited-domain activation), no Space activation and three or fewer activated indicators in total; and Type 3 (Partial multi-domain activation; residual), every remaining configuration after Types 1, 2, and 4 were assigned. In the observed dataset, all Type 3 events activated at least two domains. The fixed sequence Type 1 → Type 2 → Type 4 → Type 3 prevents overlap and assigns every configuration to one profile. Indicator-level results were displayed in a heatmap, and domain counts were visualized using radar plots. These visualizations are descriptive and do not represent severity.

## 3 Results

### 3.1 Overview of healthcare-system disruption across PHEIC events

The analysis included the eight PHEIC events declared through 31 March 2026: H1N1 influenza, poliomyelitis, Ebola virus disease in West Africa, Zika virus disease, Ebola virus disease in the Democratic Republic of the Congo, COVID-19, mpox clade II, and mpox clade I. The 12-indicator matrix showed variation in the number and configuration of threshold-level occurrences documented across events (Figure 2 and Supplementary Table 1). All events activated at least one indicator. S1 external workforce mobilization and T3 diagnostic bottleneck were documented in all eight cases. COVID-19 was the only event with all 12 indicators activated. The two Ebola events had identical binary profiles, with all Staff, Stuff, and Systems indicators activated and Space activation limited to P1. H1N1 influenza and poliomyelitis each activated five indicators; Zika virus disease three; mpox clade II two; and mpox clade I eight. Because the displayed binary matrix combines Not Activated and Not Reported, these totals describe documented activation under the coding rule rather than confirmed absence of all other occurrences.

**Figure 2:**
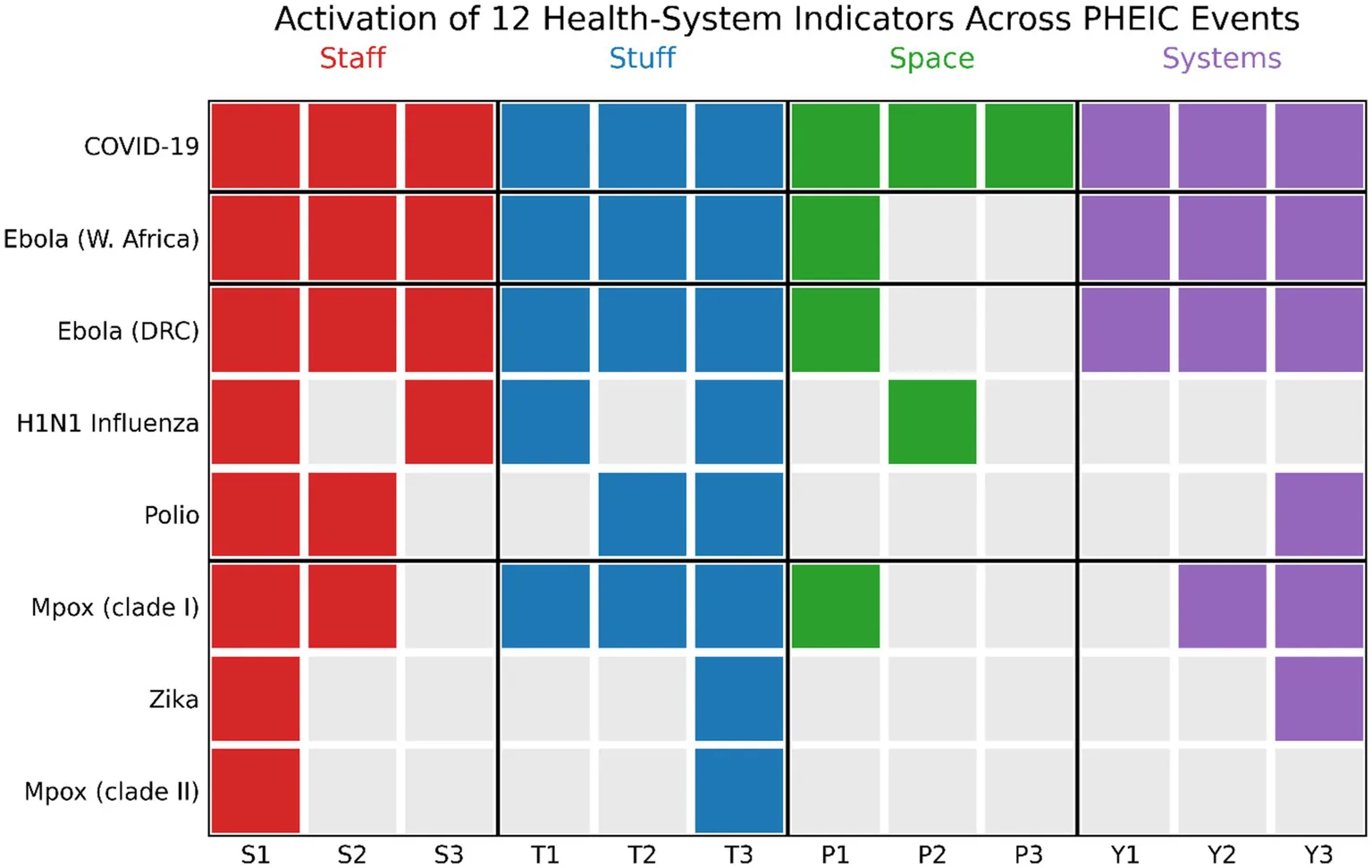
Heatmap of activated 4S indicators across eight WHO-declared PHEIC events. Colored cells indicate indicators meeting the threshold in Table 2 and identify the corresponding domain: Staff (red), Stuff (blue), Space (green), and Systems (purple). Light gray cells combine “Not Activated” and “Not Reported”; the figure does not distinguish those two extraction states. Indicators are binary and unweighted, and the figure displays documented activation patterns rather than severity, duration, geographic extent, or population-level magnitude.

Space-domain activation occurred in a subset of events. Temporary or repurposed care facilities (P1) were documented in both Ebola virus disease events, COVID-19, and mpox clade I. ICU saturation (P2) was documented in H1N1 influenza and COVID-19, and patient-transfer overflow (P3) only in COVID-19. Thus, all three predefined Space indicators were documented only for COVID-19, while selected Space indicators were documented for several other events. Systems-domain activation also varied: Y3 was documented in poliomyelitis, both Ebola events, Zika virus disease, COVID-19, and mpox clade I; Y2 in both Ebola events, COVID-19, and mpox clade I; and Y1 in both Ebola events and COVID-19.

Staff and Systems indicators partially overlapped. Healthcare worker infection disruption (S3) was documented in H1N1 influenza, both Ebola virus disease events, and COVID-19. In the Ebola events and COVID-19, S3 co-occurred with activation of all three Systems indicators. In contrast, H1N1 influenza activated S3 without Systems-domain activation, and Systems activation also occurred in events without S3 activation, including poliomyelitis, Zika virus disease, and mpox clade I. Systems activation was therefore observed both in the presence and absence of documented healthcare worker infection disruption, reflecting variable Staff–Systems co-activation patterns across events.

### 3.2 Disruption profiles across the eight PHEIC events

Activated indicators were summed within each 4S domain, yielding counts from 0 to 3. These counts describe configuration and were not interpreted as measures of severity, duration, geographic extent, or population-level impact. Domain activation profiles were visualized using radar plots (Figure 3). The eight events were assigned to four provisional descriptive profiles using the hierarchical rules specified in Section 2.6.

**Figure 3:**
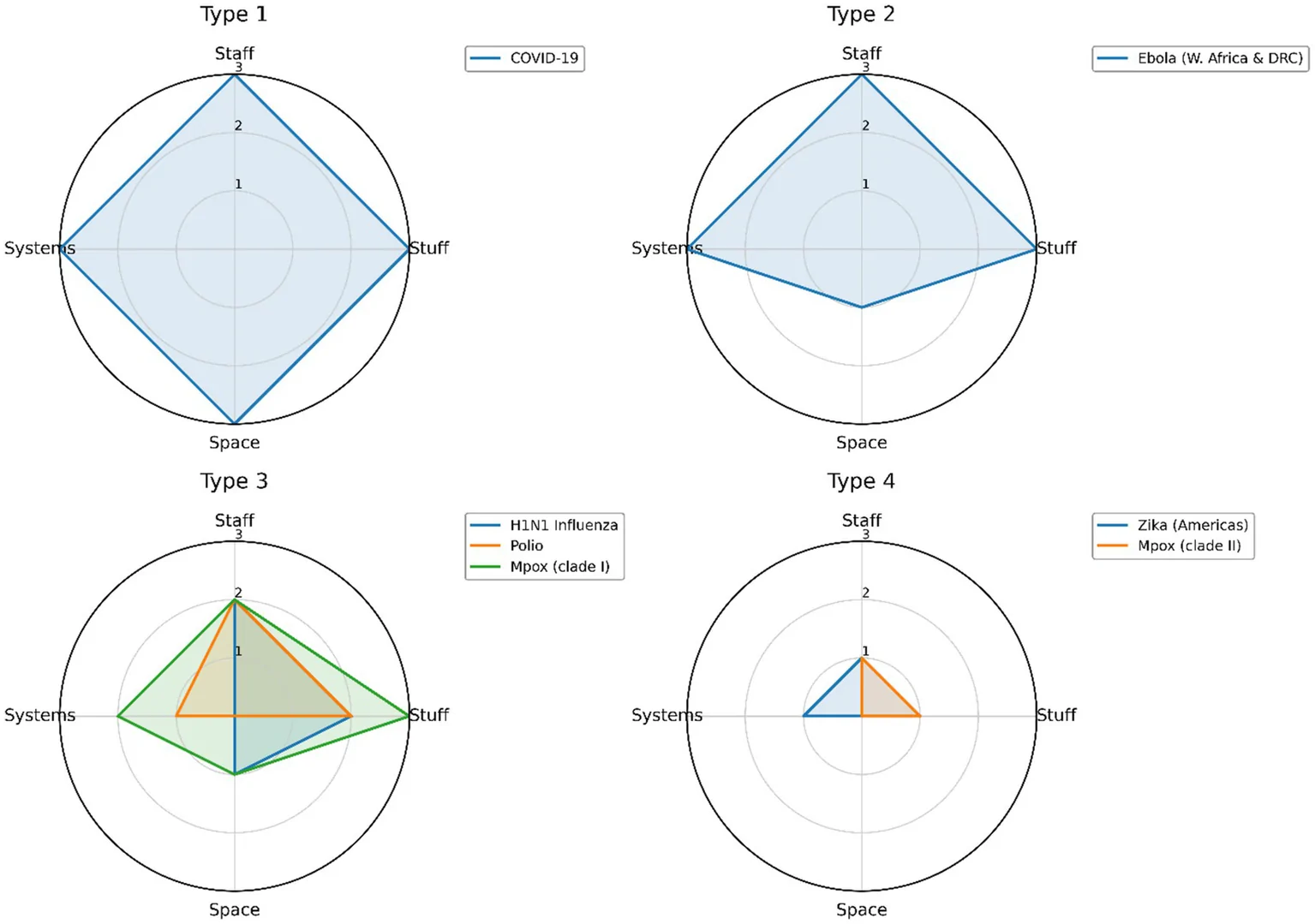
Radar plots of domain-level activation counts across the four descriptive 4S profiles. Each axis represents the number of activated indicators within one domain (Staff, Stuff, Space, and Systems), ranging from 0 to 3. Type 3 is a residual category and therefore contains heterogeneous event-specific configurations. Polygon area should not be interpreted as clinical severity, duration, geographic extent, or population-level burden.

#### 3.2.1 Type 1: systemic convergence

COVID-19 was the only event with activation of all 12 indicators, with domain counts of Staff 3, Stuff 3, Space 3, and Systems 3. This configuration reflects concurrent activation across workforce mobilization and infection disruption, material and diagnostic constraints, care-space expansion and saturation, interregional patient transfers, and routine-service restrictions.

#### 3.2.2 Type 2: workforce-cascade

Ebola virus disease in West Africa and in the Democratic Republic of the Congo had identical domain activation counts (Staff 3; Stuff 3; Space 1; Systems 3). In both events, Space activation was limited to temporary or repurposed treatment facilities (P1), without ICU saturation (P2) or structured interregional transfers (P3). Healthcare worker infection disruption (S3) co-occurred with activation of all three Systems indicators (Y1–Y3). This pattern reflects full Staff, Stuff, and Systems activation with limited Space involvement.

#### 3.2.3 Type 3: partial multi-domain activation

After Types 1, 2, and 4 were assigned, H1N1 influenza, poliomyelitis, and mpox clade I comprised the residual Type 3 category. Their domain counts were H1N1 influenza: Staff 2, Stuff 2, Space 1, Systems 0; poliomyelitis: Staff 2, Stuff 2, Space 0, Systems 1; and mpox clade I: Staff 2, Stuff 3, Space 1, Systems 2. Type 3 is intentionally heterogeneous and should not be interpreted as indicating similarity among these events beyond their failure to meet the more specific Type 1, Type 2, or Type 4 rules.

#### 3.2.4 Type 4: limited-domain activation

Zika virus disease and mpox clade II met the Type 4 rule because neither had Space-domain activation and each had three or fewer activated indicators in total. Zika virus disease activated Staff 1, Stuff 1, Space 0, and Systems 1; mpox clade II activated Staff 1, Stuff 1, Space 0, and Systems 0. In both events, S1 and T3 were activated. The classification denotes a limited number of documented indicators under the study rule and does not imply low clinical severity, successful containment, or low public-health relevance.

### 3.3 Structural features of the 4S disruption profiles

Table 3 summarizes the four profiles assigned from the 4S indicator matrix. Profiles are characterized by domain-count configurations and the hierarchical rules in Section 2.6. Domain counts do not measure disruption severity, and Type 3 is a residual rather than an internally homogeneous category.

**Table 3:** Structural features of the four 4S disruption profiles.

4S profile	Events included	Domain activation pattern	Profile definition
Type 1: Systemic convergence	COVID-19	Staff 3; Stuff 3; Space 3; Systems 3	Activation of all indicators across all four domains
Type 2: Workforce-cascade profile	Ebola virus disease, West Africa; Ebola virus disease, DRC	Staff 3; Stuff 3; Space 1; Systems 3	Full Staff, Stuff, and Systems activation with Space activation limited to temporary or repurposed treatment facilities
Type 3: Partial multi-domain activation	H1N1 influenza; poliomyelitis; mpox clade I	H1N1: Staff 2, Stuff 2, Space 1, Systems 0; Polio: Staff 2, Stuff 2, Space 0, Systems 1; Mpox clade I: Staff 2, Stuff 3, Space 1, Systems 2	Residual category assigned after Types 1, 2, and 4; in the observed dataset, all member events activated multiple domains
Type 4: Limited-domain activation	Zika virus disease; mpox clade II	Zika: Staff 1, Stuff 1, Space 0, Systems 1; Mpox clade II: Staff 1, Stuff 1, Space 0, Systems 0	No Space-domain activation and three or fewer activated indicators in total

## 4 Discussion

This study shows that, across the event-specific documentary evidence bases examined, WHO-declared PHEIC events differed in the configuration of documented health-system disruption and response. Epidemiological metrics remain essential for assessing disease burden, but they do not indicate which workforce, supply, care-space, or service-continuity functions were disrupted or reconfigured. During COVID-19, widespread cancelation and reorganization of surgical services affected essential service delivery (3). WHO guidance similarly emphasizes staff, supplies, and bed capacity in continuity planning (22), while Ebola research documents both reductions in non-target service use (23) and circumstances in which policy intervention preserved routine utilization (24). Within the present matrix, all 12 indicators were documented only for COVID-19. The comparison therefore supports a limited conclusion: the PHEIC designation did not correspond to a common 4S activation profile across the eight coded events.

The four profiles describe configurations of indicator activation rather than gradients of overall severity. Type 1 was observed only for COVID-19. H1N1 influenza had fewer predefined indicators documented, including diagnostic bottlenecks (10). Type 2 comprised the two Ebola events, which shared full Staff, Stuff, and Systems activation and P1-only Space activation. Healthcare worker infection disruption and all three Systems indicators co-occurred in these events. Reduced use of non-target health services during Ebola outbreaks has been documented in the wider literature (23). The label “workforce-cascade” is descriptive shorthand for this configuration and does not establish that workforce disruption temporally or causally produced Systems disruption.

Type 3 is the residual category after the more specific Type 1, Type 2, and Type 4 rules have been applied. Its members—H1N1 influenza, poliomyelitis, and mpox clade I—have different domain signatures and should not be treated as a coherent etiologic class. Mpox clade I combined full Stuff activation with P1-only Space activation; the present design cannot determine whether that configuration reflects event biology, baseline capacity, response intensity, observation duration, or documentation. H1N1 influenza and poliomyelitis likewise showed different selected indicators (10, 25). Type 4 indicates that three or fewer indicators were documented without Space activation; it does not demonstrate successful containment, low severity, or low public-health importance. The profiles are therefore best used as concise labels for observed configurations rather than as general properties of pathogens.

Among the events examined, PHEIC designation was not sufficient to identify a common or uniformly extensive pattern of health-system disruption. The configuration documented for an event plausibly reflects interactions among event characteristics, response choices, baseline capacity, and reporting practice. However, the 4S indicators do not measure or adjust for workforce density, laboratory networks, critical-care capacity, financing, or governance capability (26, 27). The same pathogen could therefore plausibly yield a different profile in another setting, but that proposition was not tested here. The WHO regions reported in Table 1 indicate the broad geographic settings represented in the documentary evidence base; they neither define a standardized comparison unit nor adjust for baseline structural capacity. Formal integration of baseline-capacity measures, such as IHR core-capacity or service-coverage metrics, remains a priority for future validation (26, 28).

The profiles may nevertheless be useful for generating preparedness hypotheses. A configuration involving simultaneous activation across several domains may prompt consideration of coordinated workforce, infrastructure, and supply-chain measures, as illustrated in the COVID-19 literature (29, 30). This study does not test the effectiveness of such measures or establish that a particular profile requires a specific intervention. Its contribution is narrower: it identifies which predefined domains were documented as activated and provides a structure for comparing those configurations.

For the Type 2 co-activation pattern, workforce protection is a plausible preparedness priority, but the present analysis cannot establish whether workforce disruption caused the accompanying Systems activation. Infection-prevention measures and occupational exposure monitoring have been described in prior outbreak responses (31). Likewise, vaccine-stockpile management illustrates one form of targeted response in poliomyelitis outbreaks (25). These examples should be read as possible response options from the literature rather than as interventions validated by the present typology.

This structure-oriented perspective complements pathogen-centered preparedness approaches by separating the domains in which threshold-level occurrences were documented. Operational-readiness literature similarly emphasizes workforce, supplies, space, and governance (32). The proposed typology may therefore support structured discussion of observed strain and response, but it has not been shown to improve decisions, predict escalation, or identify an optimal intervention. Prospective application and external validation are required before it is used as a decision tool.

The findings can be related cautiously to health-system resilience research, but absorptive, adaptive, and transformative capacities were not measured directly (33, 34). Type 2 documents co-activation rather than a demonstrated propagation of failure, and Type 4 documents fewer activated indicators rather than verified containment. Similarly, the repeated findings concern two specific indicators—external workforce mobilization (S1) and diagnostic bottleneck (T3)—not universal activation of all Staff and Stuff indicators. Their recurrence across eight events may motivate further study of standing workforce and diagnostic capabilities, but it does not estimate marginal investment value or justify a particular allocation strategy (35–39). One recent United States preparedness assessment provides a national example of a possible gap between written laboratory plans and the workforce or financing needed to implement them; it should not be generalized to other national systems without comparative evidence (40).

The comparison may also be relevant to international governance, provided the legal status of the instruments is stated accurately. The 2024 amendments to the International Health Regulations entered into force in September 2025 for most States Parties and introduced the “pandemic emergency” determination while reinforcing core-capacity provisions (41). The WHO Pandemic Agreement was adopted in 2025, following a multi-year negotiation directed at strengthening pandemic prevention, preparedness, and response (42). The Agreement is not open for signature until its Pathogen Access and Benefit-Sharing annex is adopted, and in May 2026 WHO Member States agreed that further negotiation of that annex was required, with the outcome to be submitted to a subsequent Health Assembly (43). The Agreement had therefore not entered into force at the time of writing, and the proposed typology does not operationalize existing Pandemic Agreement obligations. At most, it offers a provisional vocabulary that could inform future discussion of which system functions are under strain and whether response arrangements match the documented configuration. This potential link to global health security and universal health coverage remains conceptual rather than tested (27).

This study has several limitations. First, binary, unweighted indicators do not capture the intensity, duration, timing, geographic distribution, or population-level magnitude of disruption. Second, several indicators reflect adaptive response as well as strain, including external workforce mobilization, international medical teams, international material aid, and temporary facilities; activation therefore cannot be equated with system failure. Third, “Not Reported” and “Not Activated” were combined in the displayed binary matrix and typology, and no separate three-state result or missing-documentation sensitivity analysis is presented. Older or less extensively documented events may consequently appear to have fewer activations. Documentation practices also changed substantially between 2009 and 2024, creating ascertainment asymmetry that cannot be separated from true differences. Fourth, PHEIC declaration periods varied markedly, so longer events had more time and documentation opportunities in which an indicator could be observed. Fifth, the geographic scope of the documentary evidence base varied across events, from concentrated outbreak settings to multiregional documentation. Country-level comparability was therefore not achieved, and the event-level profiles may obscure substantial within-event and between-country heterogeneity. Sixth, the design was descriptive and cannot establish causal or temporal pathways between domains; the term “workforce-cascade” should be interpreted as a label for co-activation only. Seventh, the four-profile typology is a provisional author-defined partition of eight events, with Type 3 serving as a residual category, and has not been externally validated. Eighth, formal inter-rater reliability statistics were not calculated, so reproducibility beyond consensus within the study team is unknown. Ninth, the 12-indicator instrument has not undergone formal external content validation, and the three-indicators-per-domain structure was imposed for comparability rather than derived empirically. Tenth, the protocol was not prospectively registered, so the timing of indicator and profile specification cannot be independently verified. Eleventh, a complete source-to-cell audit trail for all 96 assignments is not provided, limiting independent reconstruction of individual coding decisions. Finally, baseline health-system capacity was not incorporated, so profiles are conditional on the selected setting and should not be generalized to settings with different structural starting points. The analytic cohort was closed on 31 March 2026. The subsequent PHEIC involving Ebola disease caused by Bundibugyo virus in the Democratic Republic of the Congo and Uganda, declared on 17 May 2026, therefore fell outside the scope of this analysis.

## 5 Conclusion

This study applied an author-developed 12-indicator operationalization of the established 4S framework to compare documented health-system disruption and response across eight WHO-declared PHEIC events. The event-contexts differed in their configurations of activated indicators. COVID-19 was the only event for which all 12 indicators were documented; the two Ebola events shared full Staff, Stuff, and Systems activation with P1-only Space activation; H1N1 influenza, poliomyelitis, and mpox clade I formed a heterogeneous residual Type 3 category; and Zika virus disease and mpox clade II met the limited-domain Type 4 rule. These findings show that a shared PHEIC designation did not correspond to one documented structural profile. The typology is a provisional descriptive partition, not a severity ranking, causal model, or predictive tool.

A domain-resolved description may complement epidemiological measures by specifying which predefined system functions were disrupted, constrained, or reconfigured. In this sample, S1 external workforce mobilization and T3 diagnostic bottleneck were documented in all eight events, whereas all three Space indicators were documented only for COVID-19. These frequencies generate hypotheses about recurrent preparedness needs but do not establish investment priorities. The typology may contribute a common vocabulary to discussions under the revised International Health Regulations and the developing WHO Pandemic Agreement framework, but such use requires external validation. Future research should prospectively register and test the framework, retain and report three-state coding and pre-consensus reviewer data, provide source-level auditability, incorporate duration and baseline-capacity measures, and assess temporal and within-country variation.

## Data Availability

The aggregate event-by-indicator matrix and domain activation counts are provided in Supplementary Tables 1, 2. A complete source-to-cell audit trail and pre-consensus coder-level dataset are not included in the submitted materials.
