# Supplementary material for: Healthcare system disruption across WHO-declared PHEICs: a 4S comparative case analysis
Source: Front Public Health. 2026 Sep 14;14:1901344. doi: 10.3389/fpubh.2026.1901344 (PMC13617040; doi:10.3389/fpubh.2026.1901344)
Supplement: Supplementary file 1 [file Supplementary_file_1.DOCX]

**Supplementary File 1. Detailed Search Strategy, Source Base, and Eligibility Criteria**

This supplementary file describes the documentary source base, database and grey-literature search procedures, eligibility criteria, evidentiary thresholds, and aggregate coding outputs used in the comparative analysis of eight PHEIC events.

**1. Nature of the source base**

The evidence underlying the 4S indicator coding is predominantly documentary rather than bibliographic. Indicator activation records whether a specified operational occurrence was documented—for example, a health-authority directive suspending elective surgery or the establishment of field treatment facilities. Evidence therefore came principally from WHO situation reports and Disease Outbreak News, national and subnational health-authority directives and announcements, and operational reports from international and regional agencies. Peer-reviewed observational studies were used mainly to corroborate or date occurrences identified in official documentation. Contemporaneous reporting from established news organizations was consulted only to locate underlying official material and was not used as the sole basis for activation.

This source composition follows from the event-level research question. For several events, operational records are more readily available in agency and government archives than in indexed journals, and restriction to peer-reviewed publications could preferentially represent the best-studied settings. The analysis therefore combined targeted official-document searches with database searches for supporting published evidence.

This study is a structured comparative case analysis rather than a systematic review of intervention effects. Accordingly, the supplement reports the source types, repositories, core search structure, eligibility criteria, source hierarchy, and coding thresholds used to support the comparison. It does not provide a PRISMA-style record-level accounting of all documents encountered in iterative official-archive searches.

**2. Database Search Strategy**

Structured searches were conducted in PubMed, Scopus, and Web of Science. Searches covered publications from the official declaration date of each PHEIC through March 2026.

The core Boolean structure combined event-specific terms with predefined health system keywords:

[Event-specific terms] AND (“health system capacity” OR “surge capacity” OR “workforce depletion” OR “service disruption” OR “healthcare strain” OR “hospital saturation” OR “ICU capacity” OR “diagnostic capacity”)

Event-specific terms were adapted as appropriate (e.g., “COVID-19” OR “SARS-CoV-2”; “Ebola” OR “Ebola virus disease”). Search syntax was adjusted according to database indexing requirements.

**3. Official document and grey literature search**

To capture operational documentation not indexed in bibliographic databases, targeted searches were conducted in the official repositories of:

- World Health Organization (WHO) Institutional Repository for Information Sharing (IRIS), including the Disease Outbreak News series and event-specific situation report archives
- Pan American Health Organization (PAHO)
- Africa Centres for Disease Control and Prevention (Africa CDC)
- Médecins Sans Frontières (MSF)
- National and subnational health authority websites of the jurisdictions constituting the primary analytic context for each event, searched for directives, standing orders, and official announcements issued during the declaration period

Searches combined event names with terms such as “situation report,” “technical guidance,” “response plan,” and “health service continuity.” Documents available in English, French, or Spanish were reviewed.

**4. Eligibility Criteria**

- Inclusion Criteria
- Official situation reports or technical guidance describing health system capacity, service reconfiguration, or operational strain.
- Official directives, standing orders, or announcements issued by a national or subnational health authority that establish, mandate, or report a measure corresponding to an indicator threshold in Table 2.
- Peer-reviewed observational studies reporting quantitative or qualitative evidence of healthcare disruption.
- Documents published during the official PHEIC period or in immediate post-event evaluations reporting observed system impact.
- Exclusion Criteria
- Mathematical modeling or simulation studies without empirical health system data.
- Clinical trials, laboratory studies, or animal research not addressing system-level outcomes.
- Editorials or opinion pieces lacking documented operational evidence.
- Media reporting that could not be traced to an identifiable official document or agency statement.

**5. Evidentiary requirements for coding an indicator as activated**

An indicator was coded as “Activated” only where the documentary record met one of the following conditions, applied uniformly across all eight events:

- a single primary source — a WHO situation report, an official agency report, or a health authority directive — explicitly documenting a measure or condition meeting the threshold specified in Table 2; or
- at least two independent secondary sources describing the same threshold-level condition, where no primary document was retrievable.

Where sources conflicted, priority was given to jurisdiction-specific official documents over secondary summaries. Isolated local operational adjustments were not coded as activated without evidence of regional, national, or international escalation. Two researchers independently assigned indicator-level codes, and disagreements were resolved by consensus after review of the available source material. A complete source-to-cell audit trail for all 96 event–indicator assignments is not presented in this supplement.

**Supplementary Table 1. 4S indicator activation matrix across eight WHO-declared PHEIC events.**

Indicators were coded as activated (+) when the documentary record met the threshold in Table 2 of the main manuscript. A minus sign (−) combines two extraction states: no documented activation meeting the threshold and insufficient reporting. Only activated indicators contribute to the domain counts and typology. This convention may bias less extensively documented events toward lower activation counts. Indicators are binary and unweighted and do not represent severity, duration, or population-level impact.

| **Event** | **S1** | **S2** | **S3** | **T1** | **T2** | **T3** | **P1** | **P2** | **P3** | **Y1** | **Y2** | **Y3** | **Total** |
| --- | --- | --- | --- | --- | --- | --- | --- | --- | --- | --- | --- | --- | --- |
| H1N1 Influenza | + | − | + | + | − | + | − | + | − | − | − | − | 5 |
| Poliomyelitis | + | + | − | − | + | + | − | − | − | − | − | + | 5 |
| Ebola (West Africa) | + | + | + | + | + | + | + | − | − | + | + | + | 10 |
| Zika (Americas) | + | − | − | − | − | + | − | − | − | − | − | + | 3 |
| Ebola (DRC) | + | + | + | + | + | + | + | − | − | + | + | + | 10 |
| COVID-19 | + | + | + | + | + | + | + | + | + | + | + | + | 12 |
| Mpox (Clade II) | + | − | − | − | − | + | − | − | − | − | − | − | 2 |
| Mpox (Clade I) | + | + | − | + | + | + | + | − | − | − | + | + | 8 |

Symbols: (+), Activated; (−), Not Activated or Not Reported. The displayed matrix does not distinguish the two negative states, and no separate three-state sensitivity result is provided. The Total column is the number of activated indicators out of 12 and is shown for reference only; it is not a severity score, and equal totals may represent different domain configurations.

**Supplementary Table 2. Domain activation counts and assigned disruption profile for each event.**

Domain activation counts are derived directly from Supplementary Table 1 and range from 0 to 3. Profiles were assigned hierarchically using the rules in Section 2.6 of the main manuscript: Type 1 first, Type 2 second, Type 4 third, and Type 3 last as the residual category. This sequence prevents overlap between Type 3 and Type 4 and assigns every observed configuration to one profile.

| **Event** | **Staff** | **Stuff** | **Space** | **Systems** | **Total** | **Assigned profile** |
| --- | --- | --- | --- | --- | --- | --- |
| COVID-19 | 3 | 3 | 3 | 3 | 12 | Type 1: Systemic convergence |
| Ebola (West Africa) | 3 | 3 | 1 | 3 | 10 | Type 2: Workforce-cascade |
| Ebola (DRC) | 3 | 3 | 1 | 3 | 10 | Type 2: Workforce-cascade |
| Mpox (Clade I) | 2 | 3 | 1 | 2 | 8 | Type 3: Partial multi-domain |
| H1N1 Influenza | 2 | 2 | 1 | 0 | 5 | Type 3: Partial multi-domain |
| Poliomyelitis | 2 | 2 | 0 | 1 | 5 | Type 3: Partial multi-domain |
| Zika (Americas) | 1 | 1 | 0 | 1 | 3 | Type 4: Limited-domain |
| Mpox (Clade II) | 1 | 1 | 0 | 0 | 2 | Type 4: Limited-domain |

Events are ordered by total activated indicators for presentation only; the ordering does not imply severity. Type 3 is intentionally residual and heterogeneous. Mpox clade I, H1N1 influenza, and poliomyelitis share this label because they remain after the more specific Type 1, Type 2, and Type 4 rules are applied, not because their domain configurations are assumed to be equivalent.
